# CPT1A plays a key role in the development and treatment of multiple sclerosis and experimental autoimmune encephalomyelitis

**DOI:** 10.1038/s41598-019-49868-6

**Published:** 2019-09-16

**Authors:** Anne Skøttrup Mørkholt, Michael Sloth Trabjerg, Michal Krystian Egelund Oklinski, Luise Bolther, Lona John Kroese, Colin Eliot Jason Pritchard, Ivo Johan Huijbers, John Dirk Vestergaard Nieland

**Affiliations:** 1 Department of Health Science and Technology, Aalborg University, Fredrik Bajers Vej 7, 9220 Aalborg, Denmark; 2 Mouse Clinic for Cancer and Aging Research, Transgenic Facility, The Netherlands Cancer Institute, Plesmanlaan 121, 1066 CX Amsterdam, Netherlands

**Keywords:** Multiple sclerosis, Multiple sclerosis

## Abstract

Human mutations in carnitine palmitoyl transferase 1A (*CPT1A*) are correlated with a remarkably low prevalence of multiple sclerosis (MS) in Inuits *(P479L)* and Hutterites *(G710E)*. To elucidate the role of CPT1A, we established a *Cpt1a P479L* mouse strain and evaluated its sensitivity to experimental autoimmune encephalomyelitis (EAE) induction. Since CPT1a is a key molecule in lipid metabolism, we compared the effects of a high-fat diet (HFD) and normal diet (ND) on disease progression. The disease severity increased significantly in WT mice compared to that in *Cpt1 P479L* mice. In addition, WT mice receiving HFD showed markedly exacerbated disease course when compared either with *Cpt1a P479L* mice receiving HFD or WT control group receiving ND. Induction of EAE caused a significant decrease of myelin basic protein expression in the hindbrain of disease affected WT mice in comparison to *Cpt1a P479L* mice. Further, WT mice showed increased expression of oxidative stress markers like *Nox2* and *Ho-1*, whereas expression of mitochondrial antioxidants regulator *Pgc1α* was increased in *Cpt1a P479L* mice. Our results suggest that, lipids metabolism play an important role in EAE, as shown by the higher severity of disease progression in both WT EAE and WT EAF HFD-fed mice in contrast to their counterpart *Cpt1a P479L* mutant mice. Interestingly, mice with downregulated lipid metabolism due to the *Cpt1a P479L* mutation showed resistance to EAE induction. These findings support a key role for CPT1A in the development of EAE and could be a promising target in MS treatment.

## Introduction

Multiple sclerosis (MS) is an inflammatory autoimmune disease in the central nervous system (CNS) characterized by demyelination, inflammatory plaques, oxidative stress and degradation of the blood-brain barrier (BBB)^1,2^. The etiology of MS is still not fully understood; however, different hypotheses involving infiltration of peripheral T cells activated by molecular mimicry or bystander activation, which triggers the immune system, have been proposed^3^.

Currently, most of the disease-modifying therapies on the market have shown efficacy in relapsing-remitting MS (RRMS) only, while few have shown efficacy in primary-progressive MS (PPMS) and secondary-progressive MS (SPMS)^2^. These therapies do not reverse disease development but decrease inflammation levels only^4^. MS is a heterogenic disease, and this heterogeneity underpins the challenges in developing new effective therapies that can reverse pathological damage and cure MS. The present study focused on the unique concept of dysregulated lipid metabolism as the major contributor to disease since inhibition of lipid metabolism has been effective in animal models of MS and depression^5,6^.

Genetics is important in the etiology of MS. The risk for developing MS as a child or sibling of an individual with MS is 3–5%^7^. The human leukocyte antigen (HLA)-DRB1*1501 allele is a dominant risk factor for MS with an odds ratio of 3^2,8^. Apart from mutations associated with the development of MS, mutations with protective effects against MS also exist. Several mutations in the *CPT1A* gene have been identified among humans. Two mutations in ethnic populations called Hutterites and Inuits living in northern Canada are of particular interest. The Hutterite mutation at position 2129 G to A predicts a substitution of glycine to glutamic acid at codon 710 (G710E)^9,10^ and the Inuit mutation at position 1436 C to T predicts a substitution of proline to leucine at codon 479 (P479L)^11^. These point mutations result in 0% (Hutterites) and 22% (Inuits) residual activity of the carnitine palmitoyl transferase 1 (CPT1A) protein^12,13^. The frequency of the homozygous allele mutation is 88% and 54% in Canadian and Greenland Inuits, respectively. The combined homozygous and heterozygous allele mutation frequencies are as high as 98% and 92%, respectively^11,14^. The prevalence of MS in these northern indigenous populations is remarkably low (1 per 1,100 for Hutterites and 1 per 50,000 for Inuits) compared to that in the nonindigenous population in Canada (1 per 417)^15–17^. These protective mutations indicate a significant role of CPT1A in the development of MS.

This decreased frequency of MS can be a consequence of genetics, as mentioned above, or other factors such as diet. A high-fat diet (HFD) induces brain inflammation and oxidative stress, and the consumption of HFD is associated with an increased frequency of MS and severe experimental autoimmune encephalomyelitis (EAE) disease course^18^. In contrast, restriction of caloric intake is associated with decreased inflammation in MS^18^. The traditional Inuit diet consists of animal-based diets rich in proteins and essential vitamins, suggesting that fatty acids are important modulators of inflammation as well as important energy substrates for the maintenance of energy homeostasis^19^.

Energy homeostasis in the brain is of high importance for brain function and is maintained by the glucose-fatty acid cycle^20,21^. Glucose, which is the primary energy substrate used in glycolysis and oxidative metabolism, is necessary for neuronal function, energy storage and oxidative defense^22^. Lipids are essential for the maintenance of the myelin sheath and the brain in general due to their high concentration of lipids^23^. In particular, polyunsaturated fatty acids are essential for signaling processes and membrane structure^24^. Fatty acids cross the BBB either by passive diffusion or protein-mediated transport, and the metabolism of fatty acids takes place in the mitochondria where fatty acids are converted to fatty acyl-CoA^21,25^. Fatty acyl-CoA is shuttled through the mitochondrial membrane by CPT1, converting fatty acyl-CoA into acylcarnitine, which is transported further by carnitine acylcarnitine translocase. Acylcarnitine is shuttled through the inner mitochondrial membrane by carnitine palmitoyl transferase 2 (CPT2), thereby reconverting it into carnitine and acyl-CoA used in β-oxidation^5,21^. This process underpins the rate-limiting role of CPT1 for β-oxidation, which can be reversibly inhibited by malonyl-CoA^26^.

Fatty acids are vulnerable to lipid peroxidation. Since the brain comprises a high concentration of lipids, it is particularly vulnerable to oxidative stress^27,28^. Reactive oxygen species (ROS), such as superoxide, hydrogen peroxide and hydroxyl radicals, are products of oxidative phosphorylation in the mitochondrial respiratory chain, NADPH oxidases (NOX) and monoamine oxidases^27^. When the production of these products exceeds the antioxidant capacity, the consequence is oxidative stress. In MS, ROS generated by microglia and macrophages can cause damage to myelin proteins, thus making these proteins appear similar to foreign antigens to immune cells^28^. ROS can activate transcription factors such as nuclear transcription factor-kappaβ, which upregulates the expression of tumor necrosis factor-α gene responsible for activation of immune cells^29^. Another transcription factor that is activated is nuclear factor erythroid 2-related factor (Nrf2). After activation, Nrf2 induces expression of the antioxidant enzyme heme oxygenase-1 (HO-1), thereby scavenging free radicals and removing damaged proteins^30^.

The observation of the suggestive protective mutation in *CPT1A* together with the efficacy of CPT1 inhibition in animal models of MS indicates a significant role of lipid metabolism and more specifically CPT1A in the development of CNS diseases such as MS. This hypothesis is further supported by the low levels of polyunsaturated fatty acids in MS, resulting in upregulated lipid metabolism and enhanced glucose catabolism^31^. Therefore, the purpose of this study was to clarify the role of CPT1A in MS by evaluating whether *Cpt1a P479L* mice containing the Inuit mutation are resistant to EAE, an animal model of MS.

## Results

### Generation of *Cpt1a P479L* mice

A mouse line (B6J-Cpt1a < em1Nki>) expressing the Inuit mutant allele *Cpt1a P479L* (Cpt1a <em1Nki>, MGI number: 5810634) was generated on a C57BL/6J background using CRISPR/Cas9 technology. A CRISPR-RNA guide (gRNA) that targets Cas9-mediated double-stranded DNA cleavage precisely between codons 480 and 481 of mouse *Cpt1a* (target sequence: TCCCACAGATGGCCCACGATGGG, Crispor.tefor.net specificity score: 92) was used. A 126 bp single-stranded repair oligo (top strand relative to *Cpt1a*) was ordered (Ultramer, IDT) that consisted of 60 bp 5′ homology, just 5′ of codon 479, the p.Pro479Lys (CCC > TTG) mutation and 63 bp 3′ genomic DNA homology. An injection mixture consisting of Cas9 mRNA (50 ng/µl), gRNA (25 ng/µl) and repair oligo (21 ng/µl) was prepared followed by pronuclear injection of the mixture in C57BL/6J zygotes as previously described^32^. Biopsies from F0 pups were PCR amplified using the following primers: forward CGATATAACCCTGGAAGCCCATG and reverse CTGCAGAGTTCAAGTGGGCCTG. The resulting PCR fragments were sequenced and analyzed for the desired CCC > TTG mutation. F0 mice positive for the mutation were backcrossed with C57BL/6J mice, and the F1 mice were analyzed in the same way. A mutant-specific PCR was used in subsequent generations.

Therefore, the phenotype of homozygous *Cpt1a P479L* mice was identical to that of C57BL/6J wild type (WT) mice, except for the CPT1a P479L mutation.

### The efficacy of genetic inhibition of *Cpt1a* in an EAE model

**Figure 1:**
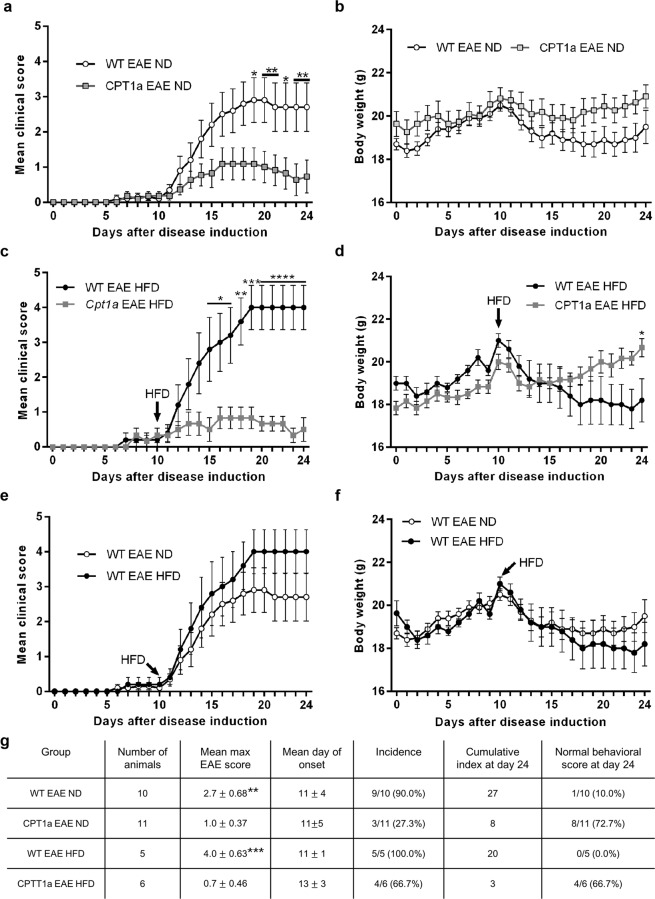
The effect of a CPT1 mutation in an EAE model in normal (ND) and high fat (HFD) diet. WT and *Cpt1a P479L* mice were immunized with MOG_35-55_ and received either ND throughout the experimental period or were introduced to HFD on the 10 day of the study. The mean clinical score was overall higher in WT EAE ND (n = 10) mice than in *Cpt1a P479L* EAE ND (n = 11) mice, and from day 19 and further, the difference between both groups was significant (**a**). No significant differences in the average body weight were observed between WT EAE ND mice and *Cpt1a P479L* EAE ND mice. However, the WT EAE ND animals were characterized with a tendency of generally lower body weight especially in the last week of the experiment (**b**). WT EAE HFD mice (n = 5) show significantly higher clinical scores compare to *Cpt1a P479L* EAE HFD mice (n = 6) from the 15 day of the experiment and onward (**c**). The weight of WT EAE HFD animals started a constant decline after introduction of HFD on day 10 of the experiment and reached significantly different level compare to *Cpt1a P479L* EAE HFD on the 24 day of the study (**d**). The comparison of WT EAE ND and WT EAE HFD animals did not reveal any significant differences. However, a clear tendency of worst clinical scores and lower body weight of HFD receiving animals was noted (**e**,**f**). Classical EAE parameter of the mean maximum score was significantly lower in *Cpt1a P479L* EAE ND mice versus WT EAE ND mice (**g**). Similarly the WT EAE HFD group was characterized with much higher mean max EAE score of 4, whereas, CPT1a EAE HFD animals appeared to be in much better condition with mean EAE score of 0.7 (**g**). Data in panels a–f analyzed with RM two-way ANOVA with Sidak’s multiple comparisons post hoc test, data in panel g analyzed with unpaired t tests. Data are presented as the mean ± SEM. Asterisks indicate the level of statistical significance (*p < 0.05, **p < 0.01, ***p < 0.001, ****p < 0.0001).

Mice were immunized with MOG_35-55_ in an EAE model of MS that lasted for 24 days. Animals were kept with free access to water and standard food representing their normal diet ND. The disease course was evaluated daily by a scale from 0 to 5 where 0 corresponds to healthy and 5 corresponds to moribund. Initially, there were no significant differences between the groups. However, from the 11 day and onwards a gradual worsening was observed in the WT EAE (n = 10) group illustrated with day-by-day increase in mean clinical sore (Fig. 1a). From 19 day of experiment the values of clinical scores for C57BL/6J WT EAE mice when compared with *Cpt1a P479L* EAE mice (n = 11) showed a pronounce and significant difference. Body weight is an important indicator of EAE progression. The daily changes in body weight between the groups varied. Although, the weight of WT EAE mice was generally lower than in *Cpt1a P479L* EAE mice it has not reached a significantly different level in the course of the experiment (Fig. 1b). The classical EAE parameters showed that *Cpt1a P479L* EAE mice had lower mean maximum EAE scores compared to WT EAE animals. Moreover, the disease incidence reached 90% for WT EAE compared to 27.3% for *Cpt1a P479L* mice (Fig. 1g).

### The effects of high-fat diet on EAE disease severity

To determine the impact of HFD on the EAE disease course, mice (WT EAE n = 5 and *Cpt1a P479L* EAE n = 6) from day 10 were introduced to HFD instead of ND up to the end of the experiment. The clinical scores of WT EAE HFD animals were significantly higher than in *CPT1a* EAE HFD from the 15 day of the experiment and onward (Fig. 1c). Importantly the significance was increasing over the following days of the experiment reaching the highly significant level (p < 0.0001) in the last six days. In terms of classical EAE parameters, the WT EAE HFD group was characterized with much higher mean max EAE score of 4, mean disease onset at day 11, incidence of 100% and cumulative index of 20. Whereas, *CPT1a* EAE HFD animals appeared to be in much better condition with mean EAE score of 0.7, mean disease onset at day 13, incidence of 66,7% and cumulative index of 3 (Fig. 1g). Noteworthy, the weight of WT EAE HFD animals started a constant decline after introduction of HFD on day 10 of the experiment. What was in contrast to *CPT1*a EAE HFD animals where an increase in the body weight was observed and finally reached significantly higher level on day 24 (Fig. 1d). However, comparison of WT EAE ND and WT EAE HFD animals did not show significant differences, but a clear tendency of worst clinical scores and lower body weight of HFD receiving animals was prominent (Fig. 1e,f).

### The effect on EAE induction on MBP and CPT1A protein expression

**Figure 2:**
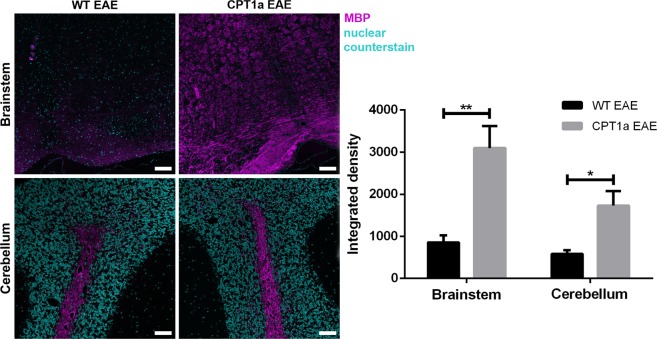
Fluorescent immunohistochemical staining of MBP with signal intensity assessment. In the brainstem, very pronounce differences in MBP labeling intensity were observed between WT EAE (n = 3) (**a**) and *Cpt1a P479L* EAE mice (n = 3). Particularly, intense signal was noted the ventral spinocerebellar tract, the pyramid and inferior olivary complexes. Labeling of these structures in WT EAE mice shown markedly weaker intensity. Similarly, in the cerebellum (illustrated on cerebral cortex, simple lobule), more intense MBP labeling was noted in the region of *arbor vitae* in the form of fibrous structures protruding into the granular cerebellar layer in *Cpt1a P479L* EAE mice. Whereas in the cerebellum of WT EAE mice, MBP labeling was noticeably less intense. Labeling intensity quantification further confirmed the observed differences in MBP expression in the brainstem and in the cerebellum between WT EAE and *Cpt1a P479L* EAE (unpaired t test) (**e**). Scale bar: 100 µm. All images acquired with identical settings at approximately −6.5 mm ant. bergma. Data are presented as the mean ± SEM. Asterisks indicate the level of significance (*p < 0.05, **p < 0.01).

MS can affect any CNS area. However, demyelination lesions are most common in the cerebellum, brainstem, periventricular white matter and spinal cord^33^. Thus, the expression of MBP was evaluated by fluorescent immunohistochemical staining of the brainstem and cerebellum in WT EAE (n = 3) and *Cpt1a P479L* EAE mice (n = 3) in both groups receiving HFD (Fig. 2). Compared to WT EAE mice, *Cpt1a P479L* EAE mice had markedly increased MBP labeling intensity with no evident pathological lesions (Fig. 2) in both the cerebellum and brainstem. In the brainstem of *Cpt1a P479L* EAE mice, most structures exhibited markedly high intensity MBP labeling in the ventral spinocerebellar tract and the pyramid and inferior olivary complexes. In contrast, in WT EAE mice, most of the medullar structures possessed low or fading labeling that slightly increased only in the ventral spinocerebellar tract and the pyramid and inferior olivary complexes. Similarly, in the cerebellum, more intense MBP labeling was noted in the *arbor vitae* in form of fibrous bundles protruding towards the granular layer in *Cpt1a P479L* EAE mice. In contrast, the characteristic MBP labeling showed noticeably lower intensity in WT EAE mice. These observations of fluorescent staining were confirmed by quantification of the labeling intensity, which resulted in significantly different values of the integrated density between the brainstem and cerebellum in both investigated groups (Fig. 2).

**Figure 3:**
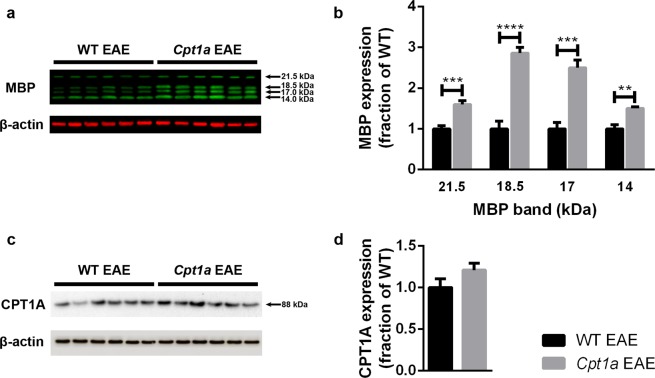
Semiquantitative immunoblotting of MBP and CPT1A. Protein expression was investigated in the HB of WT EAE (n = 6) and *Cpt1a P479L* EAE mice (n = 6). Immunoblotting with MBP revealed four bands of 14.0, 17.0, 18.5 and 21.5 kDa (**a**). All detected MBP isoforms from *Cpt1a P479L* EAE mice were expressed at significantly higher levels than those from WT EAE mice (two-way ANOVA with Sidak’s multiple comparisons post hoc test) (**b**). Analysis of CPT1A protein ex**p**ression revealed no significant differences between WT EAE and *Cpt1a P479L* EAE mice (**c**,**d**). The data were normalized to the density of the β-actin control. Data are presented as the mean ± SEM. Asterisks indicate the level of significance (**p < 0.01, ***p < 0.001, ****p < 0.0001). More information about full-length blots can be found in Supplementary Figs S1 and S2.

The protein expression of MBP was further investigated by semiquantitative immunoblotting of protein samples isolated from the hindbrain (HB) of WT EAE (n = 6) and *Cpt1a P479L* EAE mice (n = 6) (Fig. 3). Four MBP bands of 14.0, 17.0, 18.5 and 21.5 kDa were revealed (Fig. 3a). These MBP isoforms found in mice and rats arise from alternative splicing of a common mRNA precursor^34^. Importantly, all MBP isoforms in *Cpt1a P479L* EAE mice were expressed at significantly higher levels than those in WT EAE mice (Fig. 3b). The 17.0 and 18.5 kDa bands reached a nearly three-fold expression level of that found in WT EAE mice (Fig. 3b). In contrast to MBP expression, the semiquantitative immunoblotting of CPT1A of the HB of WT EAE (n = 6) and *Cpt1a P479L* EAE mice (n = 6) revealed no significant differences in the protein level (Fig. 3c,d).

### The gene expression of *Cpt1a* and *Cpt1c*

**Figure 4:**
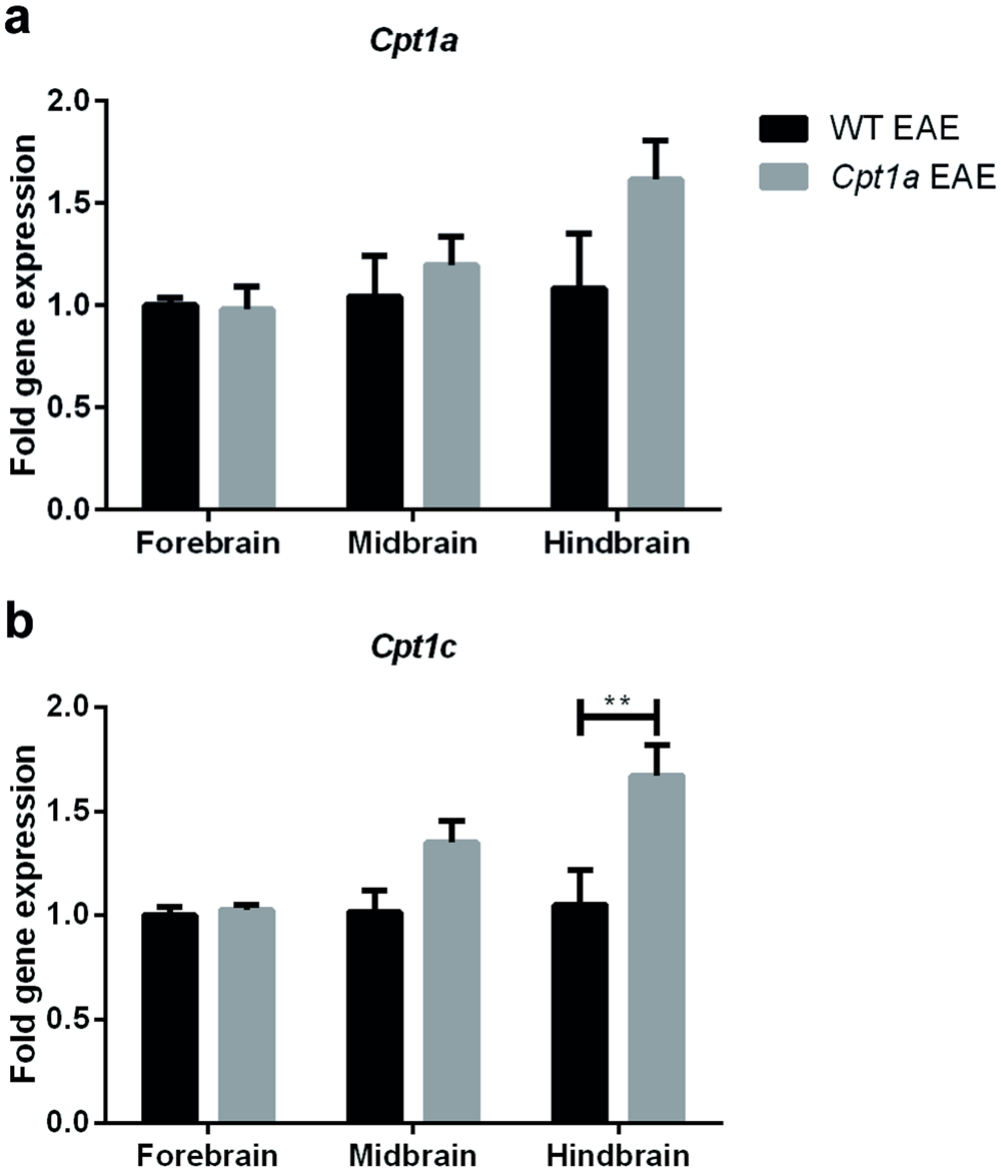
Gene expression analysis of *Cpt1a* and *Cpt1c*. The expression of *Cpt1a* (**a**) and *Cpt1c* (**b**) was investigated in the FB, MB and HB of WT EAE (n = 4) and *Cpt1a P479L* EAE mice (n = 4). No significant differences were found in the expression of *Cpt1a*. *Cpt1c* expression was significantly different in the HB between the two groups (two-way ANOVA with Sidak’s multiple comparisons post hoc test). Data are presented as the mean ± SEM. Asterisks indicate the level of statistical significance (**p < 0.01).

To evaluate whether the expression of *Cpt1a* and *Cpt1c* was affected in EAE-induced mice receiving HFD, fold gene expression was investigated by RT-qPCR in WT EAE (n = 4) and *Cpt1a P479L* EAE mice (n = 4) in both groups receiving HFD (Fig. 4). The expression was investigated in the forebrain (FB), midbrain (MB) and HB regions. There were no significant differences in the gene expression of *Cpt1a* in all three brain regions (Fig. 4a). The gene expression of *Cpt1c* in the HB showed significant differences between WT EAE mice and *Cpt1a P479L* EAE mice (Fig. 4b), whereas no changes in *Cpt1c* expression were observed in the FB and MB of the mice.

### EAE severity correlates with the gene expression of oxidative stress proteins

**Figure 5:**
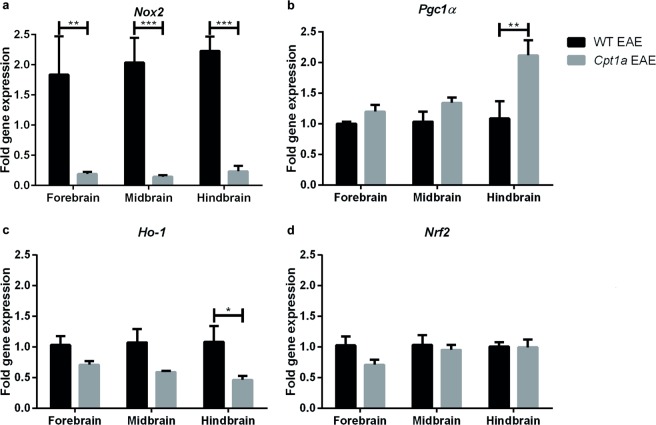
Gene expression analysis of proteins involved in oxidative stress. The expression of *Nox2* (**a**), *Pgc1α* (**b**), *Ho-1* (**c**) and *Nrf2* (**d**) in the FB, MB and HB of WT EAE (n = 3–4) and *Cpt1a P479L* EAE mice (n = 4). For statistical analysis, two-way ANOVA with Sidak’s multiple comparisons post hoc test was used. Data are presented as the mean ± SEM. Asterisks indicate the level of statistical significance (*p < 0.05, **p < 0.01, ***p < 0.001).

Oxidative stress has been described as a contributor to MS due to the high levels of lipids. Therefore, the fold change in gene expression of proteins affected by oxidative stress in the EAE model was investigated by RT-qPCR in WT EAE (n = 4) and *Cpt1a P479L* EAE mice (n = 4) in both groups receiving HFD (Fig. 5). The expression of *Nox2* was significantly decreased in *Cpt1a P479L* EAE mice compared to that in WT EAE mice in all three brain regions (Fig. 5a). The expression of peroxisome proliferator-activated receptor gamma coactivator 1 alpha (*Pgc1α*) was significantly increased in the HB in *Cpt1a P479L* EAE mice compared to that in WT EAE mice with the same tendencies observed for the FB and MB (Fig. 5b). The expression of *Ho-1* was significantly decreased in the HB in *Cpt1a P479L* EAE mice compared to that in WT EAE mice. The same tendency applied to the FB and MB; thus, no significant differences were detected (Fig. 5c). No major differences in the expression of *Nrf2* were observed (Fig. 5d).

## Discussion

For decades, MS has been considered an autoimmune disease with treatment strategies relying primarily on immunomodulatory drugs. However, this study focuses on a metabolic switch as the major contributor to disease. Therefore, the purpose of this study was to investigate the role of lipid metabolism and CPT1 in MS and in particular, the role of the *Cpt1a P479L* mutation mimicking the Inuit mutation.

MS is a disease that is characterized by a myriad of symptoms and observations. Thus far, MS has been described as an inflammatory autoimmune disease. However, current therapies focusing on the immune system have been limited to symptomatic relief in RRMS. No clinical efficacy has been gained by applying these treatments to PPMS and SPMS^2,4^. In the search for other mechanisms that could play a role in the induction and development of MS, human populations (Inuits and Hutterites) with resistance to MS were found. A few single nucleotide polymorphisms have been identified in the Inuit population where the two dominant types are in the immunoglobulin mu binding protein 2 and in CPT1A^35^. Another mutation in CPT1A was identified in the Hutterite population^9^. The mutation in the Inuit population leads to a 78% reduction in CPT1A activity^13^, whereas the Hutterite mutation results in a completely inactive CPT1A gene^9^. The Inuit mutation is present in 98% of the population living in Canada (88% homozygous and 10% heterozygous mutation) and 92% in Greenland (54% homozygous and 38% heterozygous mutation)^11^. The carrier frequency in the Hutterite population is 8%^10^. These Hutterite and Inuit people live in regions where the frequency of MS is normally high^17^. However, correlating mutations present in populations with reduced MS frequency is difficult as several other factors could be involved. Therefore, it is tempting to hypothesize that CPT1A and lipid metabolism play a key role in the development and severity of MS. For these reasons, we generated a *Cpt1a P479L* mutant mouse strain with a mutation identical to that found in the Inuit population.

Mice with the *Cpt1a P479L* mutation were tested for sensitivity to EAE induction and progression. The mice began to show EAE symptoms at day 6, and at day 12, the mean clinical score reached a score of 1, characterized by a limp tail, in the WT EAE group followed by continuous progression of disease severity indicated by a mean maximum EAE score of 2.7. These findings indicated successful EAE induction in the model. In accordance with the hypothesis, mice expressing the Inuit *Cpt1a P479L* mutation showed low mean clinical scores throughout the study after EAE induction. These scores were significantly different from those shown by WT EAE mice from day 19 to day 24. Moreover, the mean maximum EAE score in *Cpt1a P479L* EAE mice reached only a score of 1, confirming a significant reduction in disease severity compared to that in WT EAE mice. The disease incidence indicates that the *Cpt1a* mutation can ameliorate EAE symptoms. This result was further supported by the higher number of mice showing normal behavioral scores at day 24 than that of WT EAE mice. Weight loss is normally a hallmark used for evaluating the success rate of EAE induction. The body weight in the WT EAE mice started to decrease from day 10. However, insignificant the body weight in WT EAE animals represented generally lower values in comparison to their *Cpt1a P479L* EAE counterparts (Fig. 1b).

To confirm the importance of lipid metabolism in EAE and the role of CPT1A in disease induction and progression, we had introduced the HFD from the 10 day of experiment and evaluated the differences in EAE development between WT and *Cpt1a P479L* animals. The mean clinical score in WT EAE mice receiving HFD progressed significantly from day 15 up to the end of the experiment compared to *Cpt1a P479L* EAE HFD mice (Fig. 1c,d). Moreover, WT EAE HFD mice showed exacerbated disease severity, as demonstrated by their higher mean max EAE score of 4.0 versus 2.7 for WT EAE ND animals. Although, neither mean clinical score values nor the body weight of WT EAE ND and WT EAE HFE were significantly different. The overall assessment of disease progression suggest more severe EAE course among the animals challenged with HFD as was also seen by Timmermans *et al*.^18^. Additionally, our results demonstrate the resistance to EAE in *Cpt1a P479L* EAE mice receiving HFD, thus overcoming the ability of HFD to exacerbate the disease (Fig. 1c,d).

The findings of this study support our hypothesis that CPT1A and lipid metabolism play a key role in the induction and progression of MS. Herein, we demonstrated the amelioration of clinical symptoms in *Cpt1a P479L* mice in an EAE model of MS and that HFD does not affect the EAE disease course in *Cpt1a P479L* mice. This protective effect of the *CPT1a P479L* mutation could be a result of the interplay of several mechanisms affected by altered metabolism, such as inflammation, demyelination, oxidative stress and microbiota, which are all hallmarks of MS.

Inflammation, proliferation and differentiation of T lymphocytes are dependent on glycolysis and β-oxidation^36^. At the initiation of an immune response with activation of T cells, β-oxidation decreases, whereas glycolysis increases. The metabolism switches at the end of an immune response where β-oxidation is favored by memory T cells^37^. The *CPT1A* mutations found in Inuits and Hutterites are associated with increased glycolysis. In accordance with this finding, these populations are more susceptible to infections caused by stress^9,13^. However, this mutation is expected to result in milder clinical consequences in Inuits due to the residual CPT1A activity compared to the complete loss of enzymatic activity of CPT1A in Hutterites. The increased infection rate in Inuits could be caused by the hindered ability of T cells to differentiate into memory T cells as these cells are more dependent on increased β-oxidation to protect against recurring infections^38^. Pharmacological inhibition of CPT1 and β-oxidation by etomoxir downregulated inflammation in EAE animals by inducing apoptosis of activated MOG-specific T cells and reducing cytokine production^5,39^. This result was supported by less damage to myelin sheaths in etomoxir-treated EAE animals than in mice receiving placebo along with improved clinical scores (submitted for publication). Moreover, the significantly higher level of MBP expression demonstrated in this study (Figs 2 and 3) confirms a critical role of CPT1A in MS. Interestingly, the *Cpt1a P479L* mutation did not influenced neither the CPT1a gene nor protein expression level in comparison to WT animals (Fig. 3). Hence, suggesting successful introduction of Inuit mutation with only a quarter of residual activity of this mitochondrial enzyme^12,13^.

The metabolism of glucose requires less oxygen than the metabolism of lipids does. Since the findings of this study demonstrated that lipid metabolism is important in the development and severity of EAE, oxidative stress could result from the higher oxygen demand for lipid metabolism. Oxidative stress induces inflammation due to prostaglandin E2 production among other factors. This inflammatory response as well as oxidative stress is a contributor to the pathogenesis of EAE and MS due to increased production of ROS, which causes demyelination^40^. Activation of NOXs produces ROS, which are released into the intra- and extracellular space, contributing to the progression of demyelination^40^. Our gene expression analysis demonstrated significantly increased production of *Nox2* in WT EAE mice compared to that in *Cpt1a P479L* EAE mice. This finding indicates high levels of ROS in WT EAE mice that experienced oxidative stress after EAE immunization (Fig. 5). This result was also confirmed by MBP staining and intensity measurements, which were significantly reduced in WT EAE mice compared to those in *Cpt1a P479L* EAE mice, indicating degradation of myelin in WT EAE mice (Fig. 2). Since, antioxidant enzymes can scavenge ROS, we investigated the gene expression level of *Pgc1α*, a regulator of mitochondrial antioxidants peroxiredoxin-3 and thioredoxin-2 that is involved in MS pathogenesis^41^. The expression of *Pgc1α* was significantly increased in the HB in *Cpt1a P479L* EAE mice compared to that in WT EAE mice, demonstrating a defense mechanism in *Cpt1a P479L* EAE mice that restored the redox balance. The same tendencies were observed for the FB and MB in *Cpt1a P479L* EAE mice compared to those in WT EAE mice, although the differences were nonsignificant. *Ho-1*, which is also expressed in EAE, may play a protective role in the disease process^42^. Our gene expression analysis demonstrated that the expression of *Ho-1* was significantly increased in WT EAE mice compared to that in *Cpt1a P479L* EAE mice, indicating the presence of oxidative stress in WT EAE mice. Nrf2 is a regulator of cellular defense mechanisms, providing anti-inflammatory and antioxidative effects^43^. *Nrf2* is expressed in lesions in MS patients as well as in brains of EAE mice^44^; however, *Nrf2* gene expression levels were significantly altered in the EAE experiment (Fig. 5).

The role of the gut microbiota in MS has received increased attention^45^. Fatty acids can stimulate the differentiation of T cells to pathogenic Th1 and Th17 cells in the gut, resulting in inflammation in the CNS^46^. This finding means that an imbalance in the interaction of the microbiota and immune cells can cause disease and infection^47^. The traditional Inuit diet, which is an animal-based diet rich in proteins and essential vitamins, has an impact on the microbiome; nonetheless, how this factor contributes to health risk remains unclear^48,49^. Potentially, the resistance to developing EAE in *Cpt1a P479L* EAE mice could be due to modulation of the microbiota. As glucose metabolism increases, glucose levels become low and lipid levels become high throughout the body, and these metabolite balances could impact the gut microbiota.

We hypothesized that all these mechanisms proposed above might be involved in the etiology of MS. They could be initiated by stress factors, leading to hyperactivation of the hypothalamic-pituitary-adrenal axis with insulin resistance as a result. Insulin resistance leads to a shift in metabolic pathways favoring lipid metabolism, which decreases the lipid concentration and causes the demyelination of neurons. The immune system is activated by prostaglandin production and consequently causes inflammation, oxidative stress and mitochondrial dysfunction, which are all the hallmarks of MS.

The overall conclusion of this study is that the low incidence to complete absence of all types of MS corresponds well with the presence of mutation in CPT1A in the Inuit population. This finding is supported by data illustrating the resistance of *Cpt1a P479L* mice for developing an EAE-induced MS. This resistance was further supported by the findings of ameliorated clinical function, restoration of myelin and heighten antioxidant mechanisms in *Cpt1a* mutant mice. These changes support a key role of CPT1A and dysregulated, increased lipid metabolism in MS. These promising results suggest an emerging pharmacological target in the treatment of MS.

## Methods

### Genotyping of *Cpt1a P479L* mice

Mice were genotyped during breeding. DNA was extracted from ear punch tissue using a DNA kit (Zymo Research). The primer sequences used for RT-qPCR to determine the genotypes were *Cpt1a P479L* (forward: TTCCTGGGCGGACGCGTTG, reverse: CTGCAGAGTTCAAGTGGGCCTG) and WT (forward: TTCCTGGGCGGACGCGCCC, reverse CTGCAGAGTTCAAGTGGGCCTG) (TAG Copenhagen). RT-qPCR was performed using Maxima SYBR Green qPCR Master mix (Thermo Fisher). The samples were analyzed using an Agilent Aria instrument (Agilent, AH diagnostics) with the following program: 1x: 95 °C for 10 min and 40x: 95 °C for 15 s, 62 °C for 15 s, and 72 °C for 15 s.

### Animals

The animal experiments were conducted in accordance with NIH guidelines and were approved by the Danish National Committee for Ethics in Animal Experimentation (2017-15-0201-01240). C57BL/6J mice from Janvier Laboratory (Le Genest-Saint-Isle, France) and *Cpt1a P479L* mice from The Netherlands Cancer Institute were acclimated for three weeks at 21 °C in a high barrier and kept in The Animal Facility at Aalborg University. Eight-week-old female C57BL/6J mice (n = 10) and female *Cpt1a P479L* mice (n = 11) were used for EAE immunization and were receiving ND (kcal% respectively: protein 29, carbohydrate 65.5, fat 5.5, Boogarden, Denmark). For the experiment involving the effect of HFD (kcal% respectively: protein 20, carbohydrate 20, fat 60, Boogarden, Denmark; based on D12492 research diets, New Brunswick, USA) on EAE severity, 12-week-old female C57BL/6J mice (n = 5) and female *Cpt1a P479L* mice (n = 6) were used for comparison. The animals were housed in groups of five in IVC cages and maintained under standardized conditions with a 12 h light-dark cycle and ad libitum access to food and water.

### EAE immunization

C57BL/6J and *Cpt1a P479L* mice were anesthetized with isoflurane and immunized subcutaneously in the base of the tail with 200 µg MOG_35-55_ (Pepmic) emulsified in complete Freund’s adjuvant (CFA) (Becton Dickinson) containing *Mycobacterium tuberculosis* (Becton Dickinson). All mice received an intraperitoneal injection of 500 ng pertussis toxin (Sigma-Aldrich) on the day of immunization and two days later. The mice were monitored daily and weighed and scored clinically according to a scale from 0 to 5. The mice were not permitted to lose more than 20% body weight and to go beyond score 4. From day 10, the mice received HFD (60% fat) (Brogaarden). After 24 days, the mice were euthanized, and brain samples were collected for immunohistochemistry, western blotting and RT-qPCR.

### Immunohistochemical and immunofluorescent staining

Mice were anesthetized with isoflurane and perfused intracardially with 0.01 M PBS followed by fixation with 4% PFA (pH 7.4). The brains (n = 3 for WT EAE and *Cpt1a P479L* EAE) were isolated and stored in 4% PFA at 4 °C overnight. The staining procedure was performed in accordance with Oklinski *et al*.^50^. Briefly, after dewaxing and rehydration, 2 µm thick brain sections were incubated overnight at 4 °C with anti-MBP primary rabbit antibody (ab40390, Abcam) diluted 1:500. After washing, the sections were incubated with goat anti-rabbit Alexa Fluor 555 (A21422, Thermo Fisher). The cell nuclei were stained with TO-PRO®-3 (Thermo Fisher) diluted 1:1000. The sections were imaged using a Leica confocal microscope.

Images designated for fluorescent labeling intensity quantification were taken at a resolution of 1024 × 1024 pixels. A total of 10 images (five from the brainstem and five from the cerebellum) from coronal sections at approximately bregma −11 in possibly similar locations were acquired from both WT EAE and *Cpt1a P479L* EAE mice. All other microscopy settings were kept identical during the entire acquisition process. Single channel grayscale images of MBP-labeled sections were used, and fluorescent intensity was measured using ImageJ software and presented as integrated density values.

### Western blot analysis

Mouse brains (n = 6 for WT EAE and *Cpt1a P479L* EAE) were divided into the FB, MB and HB. Half of each part was collected for protein isolation used in western blotting. Protein homogenates were prepared by transferring the tissue to a dissection buffer. This mixture was then homogenized for 10 s and centrifuged at 3260 g for 18 min. The supernatant containing the cytosolic protein fraction (CPF) was saved for analysis. The pellet containing the membrane-bound and nuclear protein fraction (MBNPF) was resuspended in RIPA buffer and incubated for 30 min at 4 °C. Then, the samples were centrifuged at 6000 g for 10 min, and the supernatant was collected for analysis. The protein concentration was measured by a BCA protein assay kit (Pierce). A mixture of 10 µg CPF and 10 µg MBNPF was used for MBP blots, and 20 µg MBNPF was used for CPT1A blots.

HB protein samples were diluted in dissection buffer and sample buffer (Bio-Rad) and 5% beta-mercaptoethanol (Sigma-Aldrich) at a 1:1 ratio. Then, the samples were incubated for 15 min at 65 °C before loading separation on 4–15% Mini-Protean® TGX^TM^ Precast Protein Gels (Bio-Rad) using a Mini-Protean Tetra Cell System (Bio-Rad). After blotting, the proteins were transferred onto PVDF membranes (Bio-Rad) using a Trans-Blot Turbo Transfer System (Bio-Rad) and blocked in blocking buffer (5% skimmed milk (VWR), 0.1% Tween-20 (Sigma-Aldrich) in PBS) (PBS-T) for 1 h at room temperature. Membranes were washed and incubated overnight at 4 °C in a primary antibody solution (1% BSA in PBS-T). The primary antibodies used were MBP rat antibody (1:1000) (ab7349, Abcam), CPT1a goat antibody (1:1000) (NB100-53791, Novus Biologicals) and β-actin rabbit antibody (1:10,000) (PA5-16914, Thermo Fisher). Then, the membranes were washed and incubated in a secondary antibody solution (5% skimmed milk in PBS-T) for 1 h at room temperature. The secondary antibodies used to detect MBP and β-actin were IRDye 800CW goat anti-rat (926-32219, Li-Cor Biosciences) and IRDye 680RD goat anti-rabbit (925-68071, Li-Cor Biosciences). The secondary antibodies used to detect CPT1A and β-actin were HRP-conjugated rabbit anti-goat (P0449, Dako) and HRP-conjugated goat anti-rabbit antibody (P0448, Dako). Visualization was performed using an Odyssey Fc Imaging System (Li-Cor Biosciences) together with Li-Cor Image Studio^TM^ software. For the HRP-conjugated secondary antibodies, the blots were exposed to chemiluminescence kit detection (ECL) prior to visualization.

The density of the bands was quantified using ImageJ software. The band intensity from each lane was subtracted from the background intensity of the membrane. The labeling density values of a given protein were corrected by densitometry of the corresponding β-actin values and were normalized to facilitate comparisons. The measured band densities were pooled and expressed as the fraction of the control level (WT EAE group).

### RT-qPCR

Mouse brains (n = 4 for WT EAE and *Cpt1a P479L* EAE) were divided into the FB, MB and HB. Half of each part was collected for RNA purification using a GeneJET RNA Purification Kit (K0731, Thermo Fisher) and cDNA synthesis using a Maxima First Strand cDNA Synthesis Kit (K1671, Thermo Fisher). A quantity of 200 ng RNA was used for cDNA synthesis. The primer sequences (TAG Copenhagen) used for RT-qPCR are shown in Table 1. β-actin was used as a housekeeping control gene. RT-qPCR was performed using Maxima SYBR Green qPCR Master mix (Thermo Fisher). The samples were analyzed using an Mx3005P instrument (Agilent, AH diagnostics) with the following program: 1x: 95 °C for 10 min and 40x: 95 °C for 30 s, 60 °C for 30 s, and 72 °C for 30 s. For each sample, a nonreverse transcribed RNA sample was included as a negative control, and a water control of each primer was also included.

**Table 1:** Primer sequences used in RT-qPCR.

Gene	Forward primer	Reverse primer
β-actin	CTGTCGAGTCGCGTCCACC	TCGTCATCCATGGCGAACTGG
Cpt1a	GGAGGTTGTCCACGAGCCAG	TCATCAGCAACCGGCCCAAA
Cpt1c	CTGACCTCTGACCGGTGGGC	TTTTCCAGGAGCGCAGGGC
Ho-1	GAGCCGTCTCGAGCATAGCC	ATCCTGGGGCATGCTGTCGG
Nrf2	CGCCAGCTACTCCCAGGTTG	GGGGATATCCAGGGCAAGCG
Nox2	TGGACGGCCCAACTGGGATA	TTCAGCCAAGGCTTCAGGGC
Pgc1α	GGCTGGTTGCCTGCATGAGT	CCAACCAGAGCAGCACACTCT

### Applied statistics

Unpaired t tests, ordinary one-way ANOVA followed by Tukey’s multiple comparisons post hoc test, ordinary two-way ANOVA followed by Sidak’s multiple comparisons post hoc test, or repeated measures (RM) two-way ANOVA followed by either Tukey’s or Sidak’s multiple comparisons post hoc test were used to assess statistical significance. The fold gene expression was calculated according to the comparative C_T_ method^51^. All statistics were performed using Graph Pad Prism software. All data are presented as the mean ± SEM. P values of 0.05 were considered significant.

## Supplementary information

Supplementary Information - full blot images
