# Supplementary material for: CPT1A plays a key role in the development and treatment of multiple sclerosis and experimental autoimmune encephalomyelitis
Source: Sci Rep. 2019 Sep 16;9:13299. doi: 10.1038/s41598-019-49868-6 (PMC6746708; doi:10.1038/s41598-019-49868-6)
Supplement: Supplementary file 1 — Supplementary Information - full blot images [file 41598_2019_49868_MOESM1_ESM.pdf]

CPT1A plays a key role in the development and treatment of multiple sclerosis and experimental autoimmune encephalomyelitis

Anne Skøttrup Mørkholt[1], Michael Sloth Trabjerg[1], Michal Krystian Egelund Oklinski[1], Luise Bolther[1], Lona John Kroese[2], Colin Eliot Jason Pritchard[2], Ivo Johan Huijbers[2], John Dirk Vestergaard Nieland[1,*]

[1]Department of Health Science and Technology, Aalborg University, Fredrik Bajers Vej 7, 9220 Aalborg, Denmark

[2]Mouse Clinic for Cancer and Aging Research, Transgenic Facility, The Netherlands Cancer Institute, Plesmanlaan 121, 1066 CX Amsterdam, Netherlands

*Corresponding author: John Dirk Vestergaard Nieland, Department of Health Science and Technology, Aalborg University, Fredrik Bajers Vej 7, 9220 Aalborg, Denmark. Phone number: +45 9940 7548. Email: jdn@hst.aau.dk

<u>Supplementary figure S1 - full blot image used for preparation of Figure 3, panel a</u>

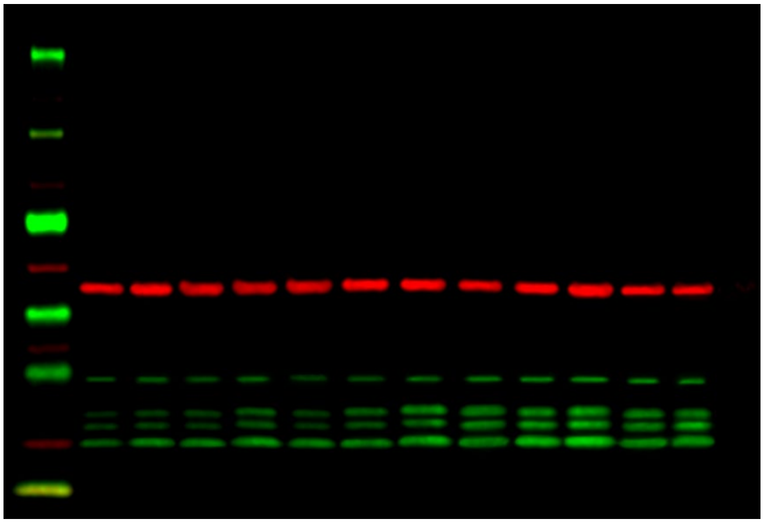

The primary antibodies used were MBP rat antibody (1:1000) (ab7349, Abcam), and β-actin rabbit antibody (1:10,000) (PA5-16914, Thermo Fisher). The secondary antibodies used to detect MBP and β-actin were; IRDye 800CW goat anti-rat (926-32219, Li-Cor Biosciences) and IRDye 680RD goat anti-rabbit (925-68071, Li-Cor Biosciences). Visualization was performed using an Odyssey Fc Imaging System (Li-Cor Biosciences) together with Li-Cor Image StudioTM software.

Supplementary Figure S2 - full blot images used for preparation of Figure 3, panel b

CPT1a blot

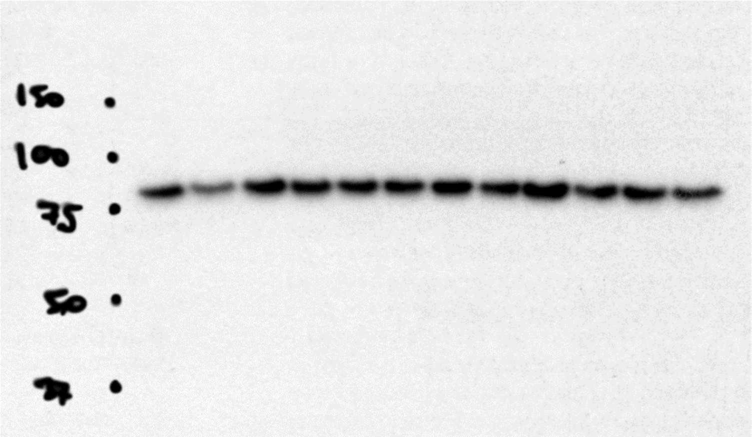

β-actin blot

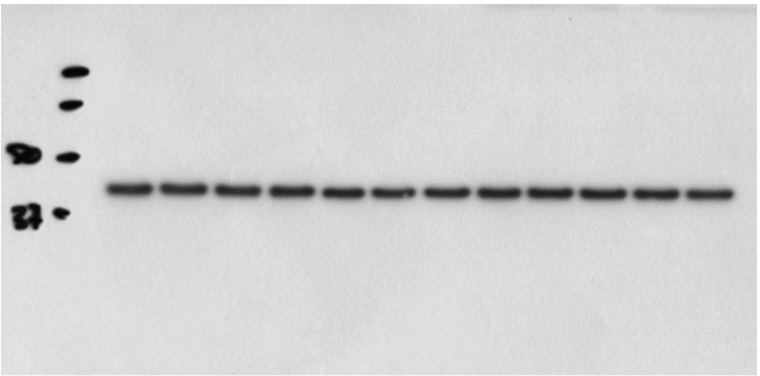

The primary antibodies used were CPT1a goat antibody (1:1000) (NB100-53791, Novus Biologicals) and β-actin rabbit antibody (1:10,000) (PA5-16914, Thermo Fisher). The secondary HRP-conjugated antibodies used to detect CPT1A and β-actin were; rabbit antigoat (P0449, Dako) and goat anti-rabbit antibody (P0448, Dako). To avoid cross-reactivity between used primary and secondary antibodies membranes for CPT1A and β-actin were incubated separately. Visualization was performed using an Odyssey Fc Imaging System (Li-Cor Biosciences) together with Li-Cor Image StudioTM. software. For the HRP-conjugated secondary antibodies, the blots were exposed to chemiluminescence detection kit (ECL) prior to visualization. Chemiluminescent marker pen WesternSure Pen (Li-Cor) was used to mark the blue protein standards from protein ladder marker to allow for molecular weight assessment.
